# (+)-Usnic Acid Induces ROS-dependent Apoptosis via Inhibition of Mitochondria Respiratory Chain Complexes and Nrf2 Expression in Lung Squamous Cell Carcinoma

**DOI:** 10.3390/ijms21030876

**Published:** 2020-01-29

**Authors:** Wanchen Qi, Changpeng Lu, Huiliang Huang, Weinan Zhang, Shaofei Song, Bing Liu

**Affiliations:** 1 School of Pharmacy, Guangdong Pharmaceutical University, Guangzhou 510006, China; 15768966527@163.com (W.Q.); 15817076492@139.com (C.L.); 18826238336@139.com (H.H.); zhangwn1996@163.com (W.Z.); songshaofei773@163.com (S.S.); 2 Guangzhou key laboratory of construction and application of new drug screening model systems, Guangdong Pharmaceutical University, Guangzhou 510006, China; 3 Key Laboratory of New Drug Discovery and Evaluation of ordinary universities of Guangdong province, Guangdong Pharmaceutical University, Guangzhou 510006, China; 4 Guangdong Key Laboratory of Pharmaceutical Bioactive Substances, Guangdong Pharmaceutical University, Guangzhou 510006, China

**Keywords:** (+)-usnic acid, lung squamous cell carcinoma, reactive oxygen species (ROS), paclitaxel

## Abstract

Lung squamous cell carcinoma (LUSC) has a poor prognosis, in part due to poor therapeutic response and limited therapeutic alternatives. Lichens are symbiotic organisms, producing a variety of substances with multiple biological activities. (+)-Usnic acid, an important biologically active metabolite of lichens, has been shown to have high anti-cancer activity at low doses. However, there have been no reports regarding the effect of (+)-usnic acid on LUSC cells. This study found that (+)-usnic acid reduced viability and induced apoptosis in LUSC cells by reactive oxygen species (ROS) accumulation. (+)-Usnic acid induced mitochondria-derived ROS production via inhibition of complex I and complex III of the mitochondrial respiratory chain (MRC). Interestingly, the elimination of mitochondrial ROS by Mito-TEMPOL only partially reversed the effect of (+)-usnic acid on cellular ROS production. Further study showed that (+)-usnic acid also induced ROS production via reducing Nrf2 stability through disruption of the PI3K/Akt pathway. The in vitro and in vivo xenograft studies showed that combined treatment of (+)-usnic acid and paclitaxel synergistically suppressed LUSC cells. In conclusion, this study indicates that (+)-usnic acid induces apoptosis of LUSC cells through ROS accumulation, probably via disrupting the mitochondrial respiratory chain (MRC) and the PI3K/Akt/Nrf2 pathway. Therefore, although clinical use of (+)-usnic acid will be limited due to toxicity issues, derivatives thereof may turn out as promising anticancer candidates for adjuvant treatment of LUSC.

## 1. Introduction

The incidence and mortality rates of lung cancer in the world are the highest among the various malignant tumors. The basic types of lung cancer can be divided into small cell lung cancer (SCLC) and non-small cell lung cancer (NSCLC), of which NSCLC accounts for about 80%–85% [1]. Lung squamous cell carcinoma (LUSC), a common histologic subtype of NSCLC, is characterized by a poor therapeutic response and poor prognosis. Unlike lung adenocarcinoma, limited therapeutic alternatives are available for LUSC [2]. Therefore, there is an urgent need to explore new effective adjuvant drugs for patients with LUSC.

Lichens are symbiotic organisms that are capable of producing a variety of substances with phenolic characteristics [3]. Usnic acid is considered as one of the most important biologically active metabolites of lichens, widely distributed in lichenized fungi of the genera Usnea, Ramalina, and Cladonia, and found in two enantiomeric forms: (−)-usnic acid and (+)-usnic acid [4]. Usnic acid has a wide range of bioactivities including antimicrobial, antiviral, anti-inflammatory, and analgesic [5]. Especially for (+)-usnic acid, it has been previously shown to have high anti-cancer activity at low doses [6]. Increasing evidence indicates that (+)-usnic acid exhibits cytotoxicity in multiple cancer cell lines, including lung adenocarcinoma cells and breast cancer cells [7,8,9]. Nevertheless, there have been no reports regarding the effect of (+)-usnic acid on LUSC cells.

Reactive oxygen species (ROS) are a class of substances produced by cellular metabolism that affect a range of signaling pathways. Cancer cells have a higher level of ROS compared with normal body cells [10]. Moderate high levels of ROS can promote the proliferation of cancer cells, while excess ROS confer toxicity on cancer cells; therefore, cancer cells are hypersensitive to ROS-inducing agents [11]. A previous study revealed that usnic acid inhibits the mitochondrial function of hepatocytes, leading to an increase in the production of ROS and inducing oxidative stress [12]. However, whether (+)-usnic acid has a similar pro-oxidative stress effect in cancer cells remains unclear. 

Herein, this study aims to investigate the potential toxicity of (+)-usnic acid in LUSC cells and the underlying mechanism. Our findings indicate that (+)-usnic acid effectively induces ROS-dependent LUSC cell apoptosis by disrupting the mitochondrial respiratory chain (MRC) and the PI3K/Akt/Nrf2 pathway. Meanwhile, (+)-usnic acid synergistically enhances the efficacy of paclitaxel in vitro and in vivo. 

## 2. Results

### 2.1. (+)-Usnic Acid Suppresses Viability and Induces Apoptosis in LUSC Cells via Cellular ROS Accumulation

**Figure 1:**
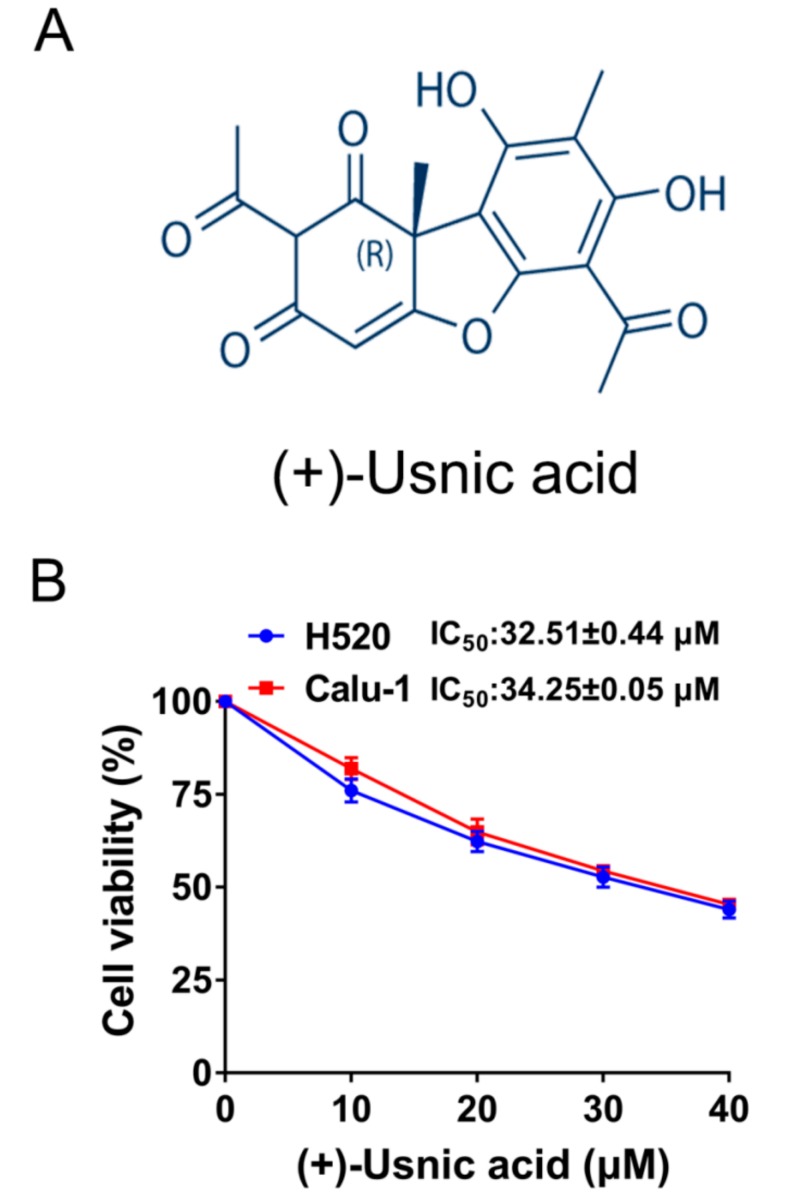
(+)-Usnic acid (UA) inhibits viability and induces apoptosis in lung squamous cell carcinoma (LUSC) cells. (**A**) The structure of (+)-usnic acid. (**B**) The effect of UA on the viability of H520 and Calu-1 cells, determined by MTT assay at the indicated concentrations after 48-hour incubation.

The structure of (+)-usnic acid with the molecular weight of 344.32 g/mol was shown in Figure 1A. Figure 1B shows that (+)-usnic acid treatment for 48 h markedly decreased the viability of two LUSC cell lines (H520 and Calu-1) in a dose-dependent manner, as determined by an thiazolyl blue tetrazolium bromide (MTT) assay, with the half-maximal inhibitory concentration (IC_50_) of 32.51 ± 0.44 to 34.25 ± 0.05 µM. 

**Figure 2:**
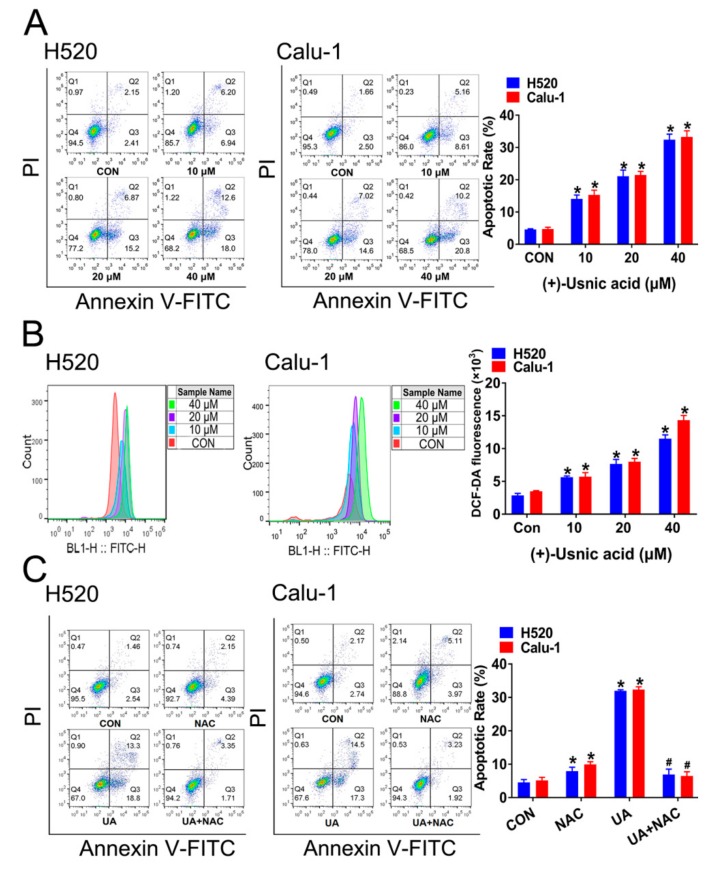
(+)-Usnic acid (UA) induces cell apoptosis via cellular ROS accumulation. (**A**) The effect of UA (10 to 40 µM) treatment for 24 h on apoptosis of H520 and Calu-1 cells. Significantly different from control group, * *p* < 0.05, *n* = 3. (**B**) The effect of UA at the indicated concentrations on ROS production in H520 and Calu-1 cells. Significantly different from control group, * *p* < 0.05, *n* = 3. (**C**) Administration of NAC (25 mM) efficiently reserved the effect of UA on cell apoptosis determined by flow cytometry. Significantly different from control group, * *p* < 0.05, *n* = 3; # significantly different from the group of UA, *p* < 0.05, *n* = 3.

To determine whether apoptosis induction contributes to inhibition of cell viability mediated by (+)-usnic acid, flow cytometry analysis was adopted. Exposure of H520 and Calu-1 cells to (+)-usnic acid led to a significant increase in apoptosis in a dose-dependent manner after 24-hour treatment (Figure 2A). Then, we sought to explore whether (+)-usnic acid confers apoptosis on LUSC cells via ROS elevation. Figure 2B shows that (+)-usnic acid dose-dependently enhanced ROS levels in H520 and Calu-1 cells after 12-hour incubation. The addition of the antioxidant *N*-acetyl-l-cysteine (NAC) (25 mM) significantly reversed the apoptotic effect of (+)-usnic acid (30 µM) on H520 and Calu-1 cells (Figure 2C). Therefore, these results strongly support that (+)-usnic acid kills LUSC cells through ROS-mediated apoptosis. 

### 2.2. (+)-Usnic Acid Damages MRC and Increases Mitochondrial ROS

**Figure 3:**
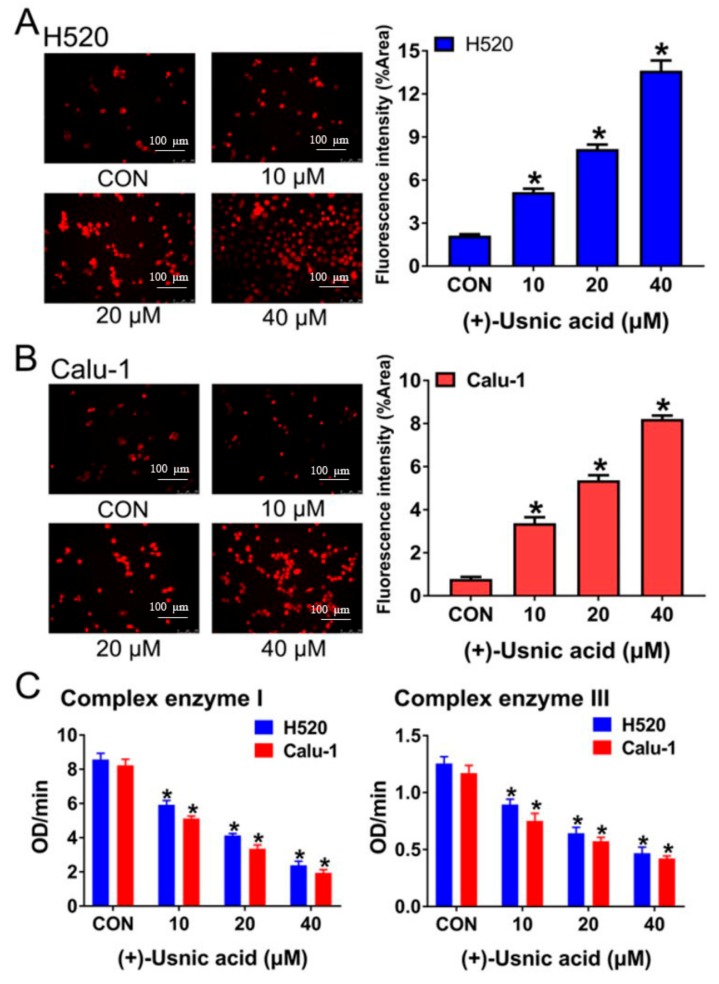
(+)-Usnic acid (UA) damages MRC and increases mitochondrial ROS. (**A**,**B**) Mito-SOX (a highly selective indicator of superoxide in live cell mitochondria) fluorescence intensity in H520 and Calu-1 cells treated with the indicated concentrations of UA detected by fluorescence microscope technique analysis. Significantly different from control group, * *p* < 0.05, *n* = 3. (**C**) Measurement of the mitochondrial complex I and III activity exposure to UA at the indicated concentrations after 12 h. Significantly different from control group, * *p* < 0.05, *n* = 3.

A previous study showed that usnic acid treatment caused early inhibition and uncoupling of the electron transport chain in mitochondria of cultured mouse hepatocytes, thus inducing oxidative stress and cell necrosis [12]. Therefore, we sought to investigate whether (+)-usnic acid-induced ROS production in LUSC cells is caused by damage to the MRC. After a 12-hour treatment of H520 and Calu-1 cells with (+)-usnic acid, cellular mitochondrial ROS were detected by a fluorescence microscope technique using a specific mitochondrial ROS probe, MitoSOX Red (5 µM). Compared with the control group, (+)-usnic acid dose-dependently enhanced mitochondrial ROS production in H520 and Calu-1 cells, reflected by the gradual increase of the red fluorescence intensity (Figure 3A,B).

In mammalian mitochondria, ROS mainly originates from NADH (ubiquinone oxidoreductase (complex I)) and ubiquinol (cytochrome c oxidoreductase (complex III)) of the electron transport chain [13]. So, we next tested the influence of (+)-usnic acid on the activity of the MRC complex enzymes I and III. As shown in Figure 3C, after incubation for 12 h, (+)-usnic acid dose-dependently damage MRC complex enzymes I and III in H520 and Calu-1 cells.

### 2.3. Interference of Nrf2 Expression Contributes to (+)-Usnic Acid-Induced ROS Production and Apoptosis in LUSC Cells 

**Figure 4:**
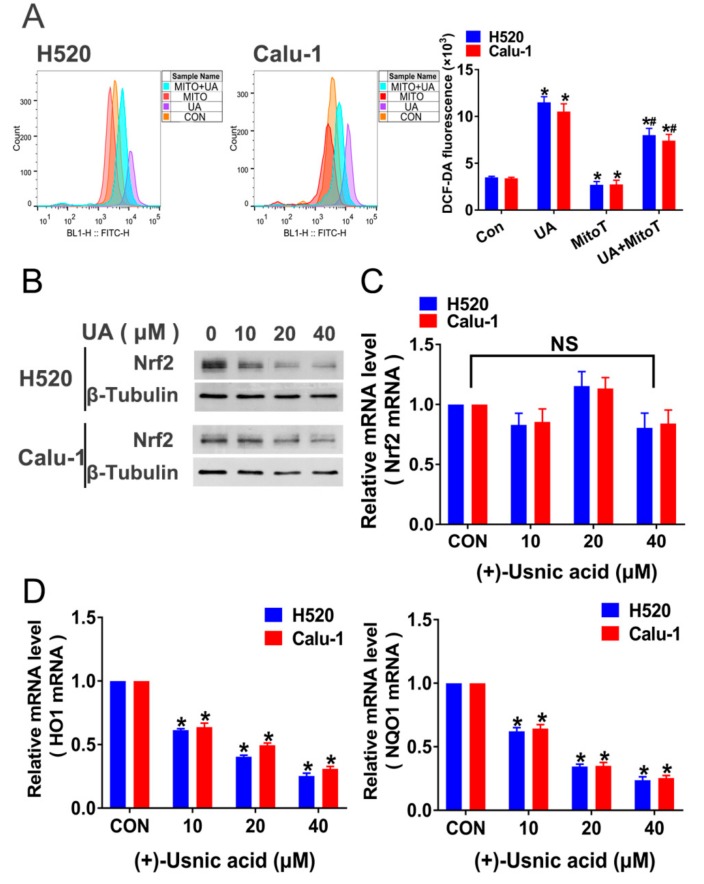
Inhibition of Nrf2 expression mediates (+)-usnic acid (UA)-induced LUSC cell apoptosis. (**A**) Administration of mitochondria-targeted antioxidant, Mito-TEMPOL (MitoT) to UA-treated H520 and Calu-1 cells. The effect of MitoT on ROS production was detected by flow cytometry. Significantly different from control group, * *p* < 0.05, n = 3; # significantly different from the group of UA, *p* < 0.05, *n* = 3. (**B**) UA (10 to 40 µM) suppressed Nrf2 expression in H520 and Calu-1 cells analyzed by Western blotting after 8-h incubation. (**C**) The effect of UA on Nrf2 mRNA expression in H520 and Calu-1 cells assayed by q-PCR. NS, not significantly, *p* > 0.05, *n* = 3. (**D**) The effect of UA on the mRNA expression of Nrf2-targeted genes (HO1 and NQO1) in H520 and Calu-1 cells determined by q-PCR. Significantly different from control group, * *p* < 0.05, n = 3.

In order to verify whether (+)-usnic acid-stimulated ROS production is specifically derived from MRC damage, we added a specific mitochondria-targeted antioxidant, Mito-TEMPOL, and detected its effect on (+)-usnic acid-induced ROS. The results show that Mito-TEMPOL (10 mM) alone efficiently eliminates ROS in H520 and Calu-1 cells; however, it only partially reversed the effect of (+)-usnic acid on cellular ROS production (Figure 4A). The above findings suggest that there exist other events involved in (+)-usnic acid-induced ROS production in LUSC cells.

To further determine the mechanism of (+)-usnic acid-induced ROS accumulation and the resulting apoptosis of LUSC cells, we then attempted to examine the effect of (+)-usnic acid on Nrf2 expression, since Nrf2 is a basic leucine zipper transcription factor encoded by the *NFE2L2* gene, regulating downstream antioxidant gene expression and playing an important role in defense against oxidative damage [14]. Figure 4B shows that (+)-usnic acid treatment for 8 h effectively inhibited the expression of Nrf2 at the protein level in a dose-dependent manner in H520 and Calu-1 cells. However, (+)-usnic acid did not affect Nrf2 mRNA expression in these cells (Figure 4C). Our further study found that (+)-usnic acid could suppress the transcriptional activity of Nrf2, as the mRNA levels of Nrf2-targeted genes, including heme oxygenase (HO1) and NAD(P)H dehydrogenase quinone 1 (NQO1), were reduced after (+)-usnic acid treatment (Figure 4D). 

**Figure 5:**
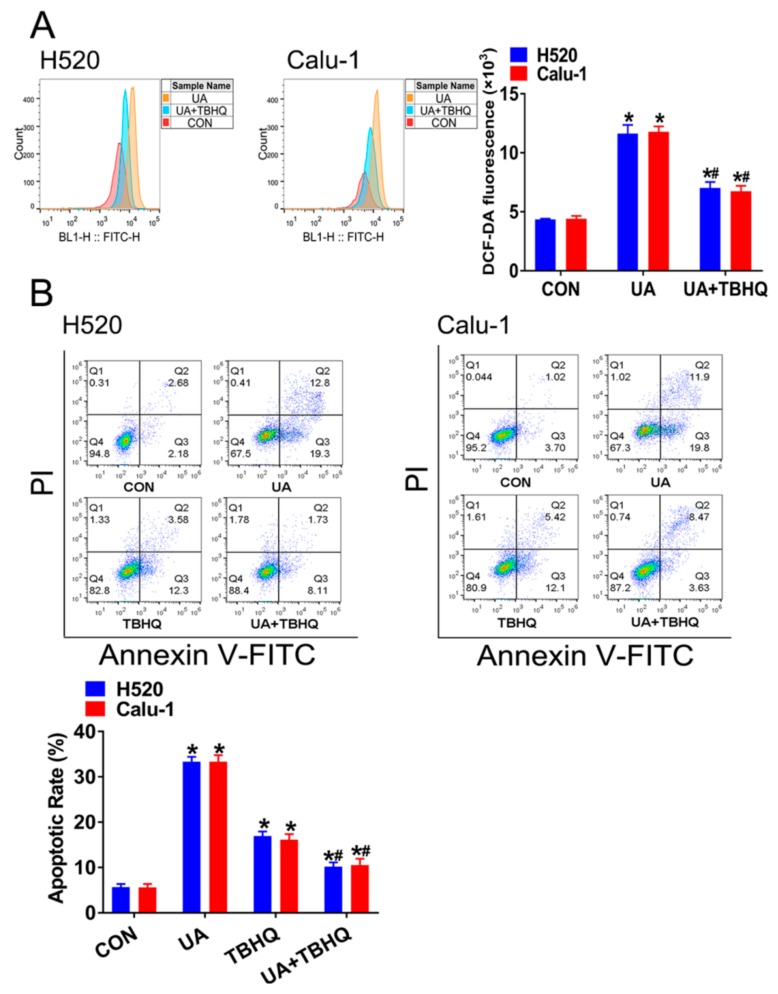
Tert-butyl hydroquinone (tBHQ) reverses (+)-usnic acid (UA)-induced H520 and Calu-1 cell apoptosis and ROS production. (**A**) TBHQ (20 µM) reversed UA-induced ROS accumulation. Significantly different from control group, * *p* < 0.05, *n* = 3; # significantly different from the group of UA, *p* < 0.05, *n* = 3. (**B**) TBHQ (20 µM) blocked UA-induced apoptosis in H520 and Calu-1 cells. Significantly different from control group, * *p* < 0.05, *n* = 3; # significantly different from the group of UA, *p* < 0.05, *n* = 3.

Tert-butyl hydroquinone (tBHQ) is a well-accepted Nrf2 agonist [15]. Next, we attempted to verify whether tBHQ can counteract the effects of (+)-usnic acid in LUSC cells. As shown in Figure 5A, treatment with tBHQ (20 µM) effectively reversed (+)-usnic acid-induced ROS accumulation in H520 and Calu-1 cells. While tBHQ alone can induce apoptosis, the addition of tBHQ to (+)-usnic acid-treated cells markedly rescued cells from (+)-usnic acid-induced apoptosis (Figure 5B). 

Therefore, these data strongly support that (+)-usnic acid induces apoptosis in LUSC cells, partially by inhibiting Nrf2 expression and accumulating ROS.

### 2.4. PI3K/Akt Signaling Mediates (+)-Usnic Acid-Inhibited Nrf2 Expression

The above finding that (+)-usnic acid decreased Nrf2 expression not at the mRNA level suggests that (+)-usnic acid interferes with Nrf2 stability. Our previous study indicated that PI3K/Akt signaling regulates Nrf2 stability in cancer cells [16] and (+)-usnic acid has been shown to have an inhibitory effect on PI3K/Akt signaling in breast cancer cells [8]. Thus, we next explored whether (+)-usnic acid inhibits Nrf2 expression via PI3K/Akt signaling in LUSC cells.

**Figure 6:**
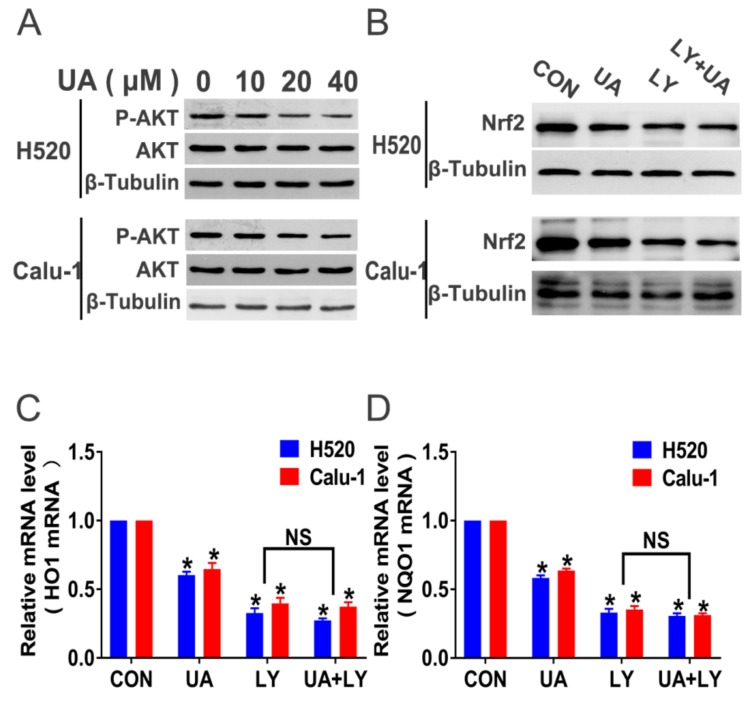
(+)-Usnic acid (UA) suppresses Nrf2 expression via inhibition of the PI3K/Akt pathway. (**A**) UA inhibited phosphorylated Akt in H520 and Calu-1 cells analyzed by Western blotting after 8-hour treatment. (**B**) After administration of LY294002 and LY294002 plus UA, the expression of Nrf2 and p-Akt was analyzed by Western blotting. (**C**,**D**) After administration of LY294002 and LY294002 plus UA, the mRNA expression of Nrf2-targeted genes (HO1 and NQO1) in H520 and Calu-1 cells by q-PCR assay. Significantly different from control group, * *p* < 0.05, *n* = 3; NS, not significantly, *n* = 3.

Figure 6A shows that (+)-usnic acid dose-dependently reduced Akt phosphorylation after 8-hour incubation in H520 and Calu-1 cells. After 8-hour administration, the selective PI3K/Akt pathway inhibitor LY294002 (30 µM) produced a similar inhibition of Nrf2 expression compared with (+)-usnic acid (Figure 6B). When the PI3K/Akt signaling was inhibited by LY294002, (+)-usnic acid treatment did not exert additional inhibition of PI3K/Akt activity compared with LY294002 alone (Figure 6B). A similar trend was also found in HO1 and NQO1 mRNA expression (Figure 6C,D). Taken together, the above data indicate that (+)-usnic acid suppresses Nrf2 expression via inhibition of PI3K/Akt signaling in LUSC cells.

### 2.5. (+)-Usnic Acid Enhances Paclitaxel Cytotoxicity

Accumulation of ROS is a key mediator for paclitaxel-induced cancer cell death [17], and we next attempted to determine whether (+)-usnic acid could sensitize LUSC cells to paclitaxel. 

**Figure 7:**
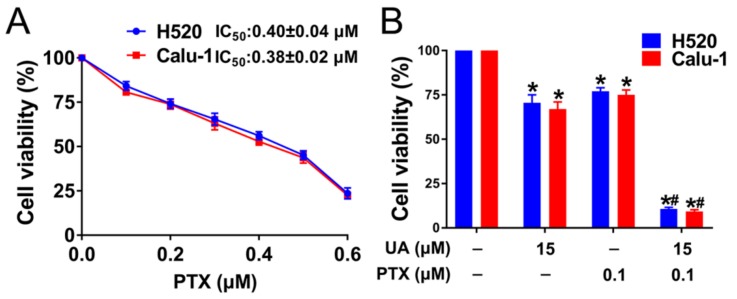
(+)-Usnic acid (UA) enhances paclitaxel cytotoxicity in LUSC cells. (**A**) Cell viability assessed by MTT after treatment with PTX at the indicated concentrations, n = 3. (**B**) The effect of UA plus PTX on H520 and Calu-1 cell viability determined by MTT assay. H520 and Calu-1 cells were exposed to different concentration of UA and PTX for 48 h. Significantly different from control group, * *p* < 0.05, *n* = 3; # significantly different from the group of UA or PTX, *p* < 0.05, *n* = 3.

Figure 7A shows that, after 48-hour treatment, paclitaxel reduced the viability of H520 and Calu-1 cells in a dose-dependent manner with IC_50_ of 0.40 ± 0.04 and 0.38 ± 0.02 µM, respectively. Then, H520 and Calu-1cells were subjected to (+)-usnic acid (15 µM) in combination with a lower dose (0.1 µM) of paclitaxel. As shown in Figure 7B, a synergistic anti-viability effect was observed in the (+)-usnic acid–paclitaxel combination compared with either agent alone. 

### 2.6. (+)-Usnic Acid inhibits Tumor Growth and Enhances Paclitaxel Efficacy in A Xenograft Mouse Model of LUSC

To determine whether these in vitro findings were applicable in vivo, female nude mice of 6-week old were subcutaneously inoculated with H520 cells (about 4 × 10^6^ cells) in the right back. When the tumor grew to 80–100 mm^3^, mice were given different treatments of (+)-usnic acid (50 mg/kg every two days for four weeks via intraperitoneal injection), (+)-usnic acid plus NAC (7 mg/ml) in drinking water during the entire experiment, paclitaxel (10 mg/kg, every two days for four weeks, by intraperitoneal injection), or (+)-usnic acid plus paclitaxel combination, respectively.

**Figure 8:**
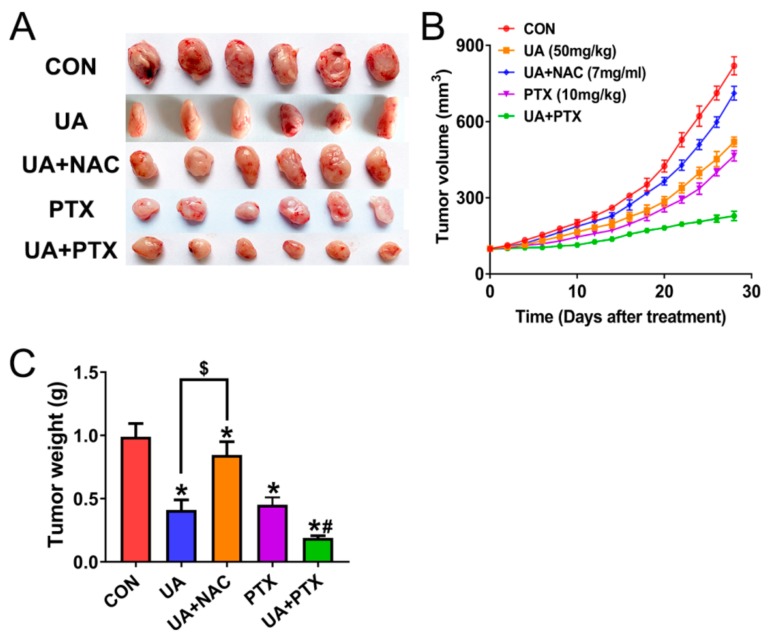
(+)-Usnic acid (UA) inhibits H520 xenograft tumor growth and enhances paclitaxel efficacy in vivo. (**A**–**C**) UA (50 mg/kg, thrice weekly, by intraperitoneal injection), UA plus the NAC (7 mg/mL given in the drinking water for the length of the experiment), PTX (10 mg/kg, thrice weekly, by intraperitoneal injection), or UA plus the PTX treatment inhibit tumor volume (**A,B**) and tumor weight (**C**). * Significantly different from control group; # significantly different from the group of UA or PTX; $ significantly different between the group of UA and UA plus NAC, *p* < 0.05, *n* = 6.

After a 28-day treatment, (+)-usnic acid and paclitaxel alone significantly inhibited tumor growth (Figure 8A–C). The use of the oxidative scavenger NAC rescued tumor inhibition caused by (+)-usnic acid. The combination of (+)-usnic acid and paclitaxel produced greater inhibition of tumor growth compared with either agent administration.

## 3. Discussion

Several studies have reported that phytochemicals (including secondary metabolites from plants) are potential sources of agents possessing anticancer activity [18,19,20]. (+)-Usnic acid (dibenzofuran derivative) is a well-known secondary metabolite found in lichens. (+)-Usnic acid has been evaluated for its anticancer potency in some cancer cell lines [6,21]; however, its efficacy in LUSC cells remains unrevealed. Herein, we evaluated the anticancer efficacy and related mechanisms of (+)-usnic acid in human LUSC cells. The findings indicate that (+)-usnic acid induces apoptosis of LUSC cells through ROS accumulation. Besides, our study also supports that a combination treatment of (+)-usnic acid and paclitaxel produces a synergistic anticancer effect in LUSC. 

It has been found that many natural products exert anticancer effects via ROS-based cell killing [22,23,24]. Normal cells usually tolerate a certain level of ROS; however, cancer cells may be more sensitive to ROS-regulating drug damage, which increases ROS levels above the redox homeostasis threshold [25]. In the present study, we found that (+)-usnic acid inhibited the viability of LUSC cells and induced apoptosis. (+)-Usnic acid increased ROS production in LUSC cells, and the elimination of ROS by its scavenger effectively rescued these cells from (+)-usnic acid-induced apoptosis. These results demonstrate that (+)-usnic acid can induce apoptosis in LUSC cells via ROS accumulation.

One of the most important sources of ROS in tumors is MRC [26], and the complexes I and III are critical sources of ROS production [27,28]. Usnic acid was reported to inhibit mitochondrial function and increase ROS production in hepatocytes [12]. Therefore, we further explored whether ROS produced by (+)-usnic acid was derived from MRC damage in LUSC. The results showed that (+)-usnic acid significantly attenuated the activity of MRC complex I and III enzymes, thereby increasing mitochondrial ROS production in LUSC cells. However, it seems interesting that targeting the elimination of mitochondrial ROS could not completely clear up the total ROS production by (+)-usnic acid. This unexpected finding strongly suggests that MRC damage is not the unique way of ROS production by (+)-usnic acid in LUSC cells. 

Nrf2 is a transcription factor, controlling cellular antioxidant responses via regulating the expression of GSH metabolism-related enzymes and enzymatic antioxidant systems and their cofactors (NADPH, FADH2) [29]. Cancer cells usually thrive under high oxidative stress due to constitutive activation of Nrf2, implying that Nrf2 could be a good and promising target against cancer [30]. In this study, we found that (+)-usnic acid effectively inhibited the expression of Nrf2 in LUSC cells, and activation of Nrf2 by its activator reversed the ROS accumulation and cell apoptosis induced by (+)-usnic acid. These results indicate that inhibition of Nrf2 is also critically involved in the (+)-usnic acid-induced ROS burst and resultant LUSC cell apoptosis. 

This study found that (+)-usnic acid reduced Nrf2 expression at the protein level but not the mRNA level, suggesting that (+)-usnic acid may interfere with Nrf2 stability in LUSC cells. Nrf2 stability is tightly regulated by Kelch-like ECH-associated protein 1 (Keap1), which targets Nrf2 for its proteasomal degradation, and glycogen synthase kinase-3β (GSK-3β) [31]. Since GSK-3β is negatively regulated by the PI3K/Akt axis via phosphorylation at Serine 9 (Ser 9) [32], and (+)-usnic acid was shown to inhibit Akt activity in breast cancer cells [33], we speculated whether (+)-usnic acid interferes with Nrf2 expression via the PI3K/Akt pathway in LUSC cells. Our results indicate that (+)-usnic acid reduced Akt phosphorylation of Ser 9 in H520 and Calu-1 cells. Inhibition of the PI3K/Akt pathway exerted a similar inhibitory effect on Nrf2 expression with (+)-usnic acid, and when PI3K/Akt signaling was blocked, (+)-usnic acid treatment did not exert additional inhibition of Nrf2 expression in LUSC cells. These data strongly support that (+)-usnic acid regulates Nrf2 expression mainly in a PI3K/Akt-dependent manner in LUSC cells. 

Accumulation of ROS by paclitaxel contributes largely to its cytotoxicity in cancer cells, and inhibition of Nrf2 enhances its efficacy in cancer cells. In this study, we found that combining (+)-usnic acid and paclitaxel resulted in synergistic effects on viability inhibition in LUSC cells. Besides, the in vivo data supported that (+)-usnic acid was efficient to reduce H520 tumor, and the combination treatment of (+)-usnic acid and paclitaxel was even more effective than either single-drug treatment. Therefore, the treatment of (+)-usnic acid may be effective in a clinical setting to enhance chemosensitivity of paclitaxel in LUSC. 

In conclusion, this study demonstrates that (+)-usnic acid induces ROS accumulation and cell apoptosis, as well as enhances the antitumor efficacy of paclitaxel in LUSC cells by disrupting the MRC and interfering with Nrf2 expression via inhibition of the PI3K/Akt pathway. Therefore, although the clinical use of (+)-usnic acid will be limited due to toxicity issues, derivatives thereof may turn out as promising anticancer candidates for adjuvant treatment of LUSC. 

## 4. Materials and Methods 

### 4.1. Materials

(+)-Usnic acid (S2252) was purchased from Selleckchem (SH, China). Tert-butylhydroquinone (tBHQ) (HY-B0015, CAS Number: 33069-62-4), LY294002 (HY-10108, CAS Number: 154447-36-6) and paclitaxel (HY-B0015, CAS Number: 33069-62-4) were purchased from MedChemExpress Co. (SH, China). *N*-acetyl-l-cysteine (NAC) (#A7250, CAS Number: 616-91-1) was obtained from Sigma Chemical Co. (SH, China). All other reagents were from Sigma Chemical Co. unless otherwise stated.

### 4.2. Cell Lines and Culture

Human LUSC H520 and Calu-1 cell lines were originally from ATCC (Manassas, VA, USA). Cells were grown in Roswell Park Memorial Institute (RPMI)-1640 media supplemented with 10% FBS (GIBCO, Invitrogen, USA) and in a humidified atmosphere under 5% CO_2_ and 37 °C temperature in a CO_2_ incubator (Thermo Fisher Scientific, Waltham, MA, USA).

### 4.3. Cell Viability Assay

The protocol used for the MTT assay was strictly according to our previous study [34]. Cells were seeded into 96-well plates at a density of 5 × 103 cells/well in 100 µL of culture medium and incubated for 48 h with individual treatment. Then, about 100 µL of fresh media and 20 µL of MTT solution were added to each well and cells were incubated for 4 h. Supernatants were carefully removed and formazan crystals were dissolved in 150 µL of dimethyl sulfoxide (DMSO). The plates were incubated for 10 min with gentle shaking before measuring the absorbance at 570 nm using a microplate reader (Thermo Fisher Scientific Multiskan™ FC, USA). IC50 values were calculated using GraphPad Prism v. 7.01 (GraphPad Software, San Diego, CA, USA).

### 4.4. Apoptosis Determination

After 24-hour individual treatment, cells were trypsinized and collected, and then washed twice with cold PBS. Cells were resuspended in 400 µL 1× binding buffer with 5 µL Annexin V-FITC and 10 µL propidium iodide (PI) (BB-4101-3). After incubation for 15 min at 4 °C in the dark and analyzed by flow cytometry (Thermo Fisher Scientific, Attune NxT, USA).

### 4.5. ROS Detection

Intracellular ROS level was detected by staining cells with 2,7-dichlorofluorescein diacetate (DCF-DA, Sigma Chemical Co., CAS Number: 35845). Briefly, cells were trypsinized and collected by centrifugation, then washed twice by PBS and stained with 10 µM DCF-DA in Hank’s balanced salt solution (HBSS) for 30 min. The stained cells were washed with PBS and analyzed by flow cytometry (Thermo Fisher Scientific, Attune NxT, USA) with an excitation wavelength of 488 nm and an emission wavelength of 525 nm.

### 4.6. Measurement of Mitochondrial Superoxide

Mitochondria-derived ROS was detected with the mitochondrial superoxide indicator MitoSOX-Red (Molecular Probes Inc., Eugene, OR, USA). The cells were harvested, washed twice in PBS, and incubated with 5 µM MitoSOX-Red for 15 min at 37 °C, followed by analysis on a fluorescence microscope (Zeiss Axio observer, Z1, German).

### 4.7. Enzymatic Activity of The Electron-Transport-Chain Components

Complex I activity was assayed with the Complex Human Enzyme Activity Microplate Assay kit (Abcam) according to the manufacturer’s instructions. Complex III activity was assayed with the Mitochondrial Complex III Activity Detection kit (GENMED, SH, China).

### 4.8. Western Blotting

Cells were harvested and lysed (Cell Signaling Technology, Beverly, MA, USA) after treatment with a separate drug at the specified time with 0.5% protease inhibitor cocktail (Sigma-Aldrich, St Louis, MO, USA). The membranes were first probed with primary antibodies as follows: anti-Nrf2 antibody (Abcam Cat#ab31163, RRID: AB_881705, Cambridge, USA), anti-p-Akt antibody (Cell signaling Cat#4060, RRID: AB_2315049, USA), anti-Akt antibody (Cell signaling Cat#4685, RRID: AB_2225340, USA), and anti-β-tubulin antibody (Abcam Cat# ab6046, RRID: AB_2210370, Cambridge, USA). For analysis of Nrf2 and p-Akt, blots were probed with their specific antibodies (diluted with 5% BSA to 1:1000). For analysis of β-tubulin, blots were probed with its antibody (diluted with 5% BSA to 1:5000). Membranes were probed with horseradish peroxidase (HRP)–labeled anti-rabbit secondary antibody from Cell Signaling (diluted with 5% BSA to 1:1000, USA). Antibody binding was detected by an enhanced chemiluminescence detection kit (ECL) (UK Amersham International plc, UK).

### 4.9. Real-Time RT-PCR

Total RNA was extracted from H520 and Calu-1 cells using Trizol reagent (Invitrogen), and then complementary DNA (cDNA) was synthesized using a ReverTra Ace reverse transcriptase (Japan TOYOBO, FSQ-301, Japan) according to the manufacturer's protocol. Real-time RT-PCR was performed with the SYBR Green Realtime PCR Master Mix (TOYOBO, Japan, QPK-201) on an iCycler (Bio-Rad, OSA, Japan) following the manufacturer’s instructions. The primer sequences were as follows: Nrf2 forward primer: 5′-GACGTGTGGCGGCTGAGC-3′; Nrf2 reverse primer: 5′-GCACCGCGTCCGAACTAGAAG-3′; GAPDH forward primer: 5′-GGCACCGTCAAGGCTGAGAAC-3′; GAPDH reverse primer: 5′-CATGGTGGTGAAGACGCCAGTG-3′; HO-1 forward primer: 5′-GGTGCTCGTACTGCTACTGTCATG-3′; HO-1 reverse primer: 5′-GCCACGAACCTCATCTCTTCCAC-3′; NQO-1 forward primer: 5′-CGCCTGCCATCATGCCTGAC-3′; NQO-1 reverse primer: 5′-GTGTGGTGGATCACGCCTGTAATC-3′. The gene expression levels for each amplification were calculated using the ΔΔCT method and normalized against GAPDH mRNA.

### 4.10. Xenograft Models

The animal experiment and procedures were approved by the Animal Center of Guangdong Pharmaceutical University (GDPU20190193, 2019/6/2). All animal experiments complied with the National Institutes of Health guide for the care and use of laboratory animals (NIH Publications No. 8023, revised 1978). Female athymic nude mice (4–6 weeks old) were purchased from the Guangdong Medical Laboratory Animal Center.

H520 cells (approximately 4 × 10^6^ cells) were subcutaneously inoculated into the right flank of 6-week-old female nude mice. When the tumors had achieved to about 80–100 mm^3^, the mice were divided into five groups (*n* = 5 per group). The mice were differently treated with (+)-usnic acid (50 mg/kg, once two days, by intraperitoneal injection), (+)-usnic acid plus NAC (7 mg/mL given in the drinking water for the length of the experiment), paclitaxel (10 mg/kg, once two days, by intraperitoneal injection), or (+)-usnic acid plus paclitaxel, respectively. The therapy was continued for 4 weeks. The tumor weight and size were measured and calculated once two days. The mice were sacrificed 4 weeks after the treatment.

### 4.11. Statistical Analysis

All in vitro experiments were performed in triplicate. Pooled data were subjected to statistical analyses using GraphPad Prism v. 7.01 (GraphPad Software, CA, USA). Differences between means from two different groups were subjected to Student’s *t*-tests, whereas one-way analysis of variance (ANOVA) was used to test for significant differences between three or more groups. The in vivo tumor growth data were subjected to two-tailed Student’s *t*-tests. Results were considered to be significantly different when *p* < 0.05, indicated by * or # symbols.

## Abbreviations

DCF-DA	2,7-Dichlorofluorescein diacetate
DMSO	Dimethyl Sulfoxide
FA–CI	Fraction-effect versus combination index
FBS	Fetal bovine serum
HO1	Heme oxygenase 1
GSK-3β	Glycogen synthase kinase-3β
Keap1	Kelch-like ECH-associated protein 1
LUSC	Lung squamous cell carcinoma
MitoT	Mito-TEMPOL
MRC	Mitochondrial respiratory chain
MTT	3-(4,5-dimethyl-2-thiazolyl)-2,5-diphenyl-2-H-tetrazolium bromide, Thiazolyl Blue Tetrazolium Bromide
NAC	N-acetyl-L-cysteine
NQO1	Dehydrogenase, quinone 1
NSCLC	Non-small cell lung cancer
PBS	Phosphate buffered solution
PI	Propidium iodide
ROS	Reactive oxygen species
(RPMI)-1640	Roswell park memorial institute
tBHQ	Tert-butyl hydroquinone
