# Correction: Qi, W., et al. (+)-Usnic Acid Induces ROS-Dependent Apoptosis via Inhibition of Mitochondria Respiratory Chain Complexes and Nrf2 Expression in Lung Squamous Cell Carcinoma. *Int. J. Mol. Sci.* 2020, *21*, 876

**DOI:** 10.3390/ijms21082915

**Published:** 2020-04-21

**Authors:** Wanchen Qi, Changpeng Lu, Huiliang Huang, Weinan Zhang, Shaofei Song, Bing Liu

**Affiliations:** 1 School of Pharmacy, Guangdong Pharmaceutical University, Guangzhou 510006, China; 15768966527@163.com (W.Q.); 15817076492@139.com (C.L.); 18826238336@139.com (H.H.); zhangwn1996@163.com (W.Z.); songshaofei773@163.com (S.S.); 2 Guangzhou Key Laboratory of Construction and Application of New Drug Screening Model Systems, Guangdong Pharmaceutical University, Guangzhou 510006, China; 3 Key Laboratory of New Drug Discovery and Evaluation of ordinary universities of Guangdong province, Guangdong Pharmaceutical University, Guangzhou 510006, China; 4 Guangdong Key Laboratory of Pharmaceutical Bioactive Substances, Guangdong Pharmaceutical University, Guangzhou 510006, China

The authors wish to make the following corrections to this paper [1]:

Figure 2 contains some errors and it should be replaced with the correct figure. 

During the initial submission, the authors submitted the wrong Figure 2. The authors performed flow cytometry assay for many repetitions. At first, the authors adjusted the flow cytometry after the analysis of the control sample in order to confine the normal cell subgroup more precisely, however, they carelessly saved these “duplicated” images (Figure 2C: H520 panel, CON group and Calu-1 panel, CON group vs. Figure 2A: Calu-1 panel, CON group). The authors have provided the correct images (H520 panel, CON group and Calu-1 panel, CON group) of Figure 2C. The authors have also provided all the original fcs files of flow cytometry images. 

The authors would like to apologize for any inconvenience caused to the readers by these changes.
